# Evaluation of microplate immunocapture method for detection of *Vibrio cholerae*, *Salmonella* Typhi and *Shigella flexneri* from food

**DOI:** 10.1186/s12866-017-1099-y

**Published:** 2017-08-29

**Authors:** Md. Fakruddin, Md. Nur Hossain, Monzur Morshed Ahmed

**Affiliations:** Industrial Microbiology Laboratory, Institute of Food Science and Technology (IFST), Bangladesh Council of Scientific and Industrial Research (BCSIR), Dhaka, 1205 Bangladesh

**Keywords:** Immunocapture, Microplate, Enrichment, Detection, Pathogen, Food

## Abstract

**Background:**

Improved methods with better separation and concentration ability for detection of foodborne pathogens are in constant need. The aim of this study was to evaluate microplate immunocapture (IC) method for detection of *Salmonella* Typhi, *Shigella flexneri* and *Vibrio cholerae* from food samples to provide a better alternative to conventional culture based methods.

**Results:**

The IC method was optimized for incubation time, bacterial concentration, and capture efficiency. 6 h incubation and log 6 CFU/ml cell concentration provided optimal results. The method was shown to be highly specific for the pathogens concerned. Capture efficiency (CE) was around 100% of the target pathogens, whereas CE was either zero or very low for non-target pathogens. The IC method also showed better pathogen detection ability at different concentrations of cells from artificially contaminated food samples in comparison with culture based methods. Performance parameter of the method was also comparable (Detection limit- 25 CFU/25 g; sensitivity 100%; specificity-96.8%; Accuracy-96.7%), even better than culture based methods (Detection limit- 125 CFU/25 g; sensitivity 95.9%; specificity-97%; Accuracy-96.2%).

**Conclusion:**

The IC method poses to be the potential to be used as a method of choice for detection of foodborne pathogens in routine laboratory practice after proper validation.

## Background

Foodborne pathogens are a growing concern for human illness, death, and food safety and security [1]. The analysis of foods for pathogen presence is a standard practice for ensuring the safety of food, identifying agents of foodborne illness and determining sources of foodborne outbreaks. Conventionally, the microbiological analysis of food involves culture enrichment followed by isolation on selective media [2]. The initial pre-enrichment of a food sample allows for resuscitation of physiologically stressed microbes and grows all bacteria to detectable levels (>10 CFU/ml), followed by a period of selective enrichment to enable growth of the target organism. From there, the pathogen, if present, is isolated on selective agar, and purification and confirmation occur using morphological, biochemical, or physiological tests [3]. Conventional culture methods, however, are often problematic, in that many are time-consuming and require several days to complete, appropriate selective media are not currently available for all bacterial foodborne pathogens, some bacterial pathogens require specific atmospheric or other growth conditions which may be difficult to simulate in the laboratory and some bacterial pathogens may not be culturable by currently available methods [4]. The presence of high background indigenous microflora and complex matrix in food also limits detection of pathogen from food samples. Again, pathogen often exist in viable but non-culturable states in food which cannot be detected by the conventional culture based method [5]. There is a great need for improved methods for foodborne pathogen detection in food matrices. Concentration and separation of pathogens from the food matrix has been the focus of many studies investigating ways to improve sample assay detection limits and speed time to results.

Immunocapture (IC) to concentrate target pathogen from food samples offers a better alternative to traditional pre-enrichment and enrichment steps for routine analysis in microbiology laboratories [6, 7]. Antibody attached to solid surface can capture bacteria through attachment to cell surface proteins and allow specific isolation of the bacteria from samples with high background flora [8]. IC can be used in combination with culture or molecular methods for isolation and detection of the pathogens. One form of IC, immune-magnetic separation (IMS) (that uses magnetic beads coated with antibody) has been developed against many pathogens such as *Salmonella* spp. [9], *Listeria monocytogenes* [10], *E. coli* O157:H7 [11] and *Vibrio parahaemolyticus* [12] and is commercially available. IMS has been investigated for the concentration and purification of bacterial pathogens from food samples [13] and are reportedly more sensitive than comparable conventional culture methods [14]. By using IMS, PCR inhibitors inherent to fecal samples were successfully eliminated [15], but this approach is limited in routine laboratories of underdeveloped countries due to instrumentation cost.

This study focused on three important foodborne pathogens: *Vibrio cholerae*, *Salmonella* Typhi and *Shigella flexneri*. Many previous reports have showed prevalence of *Vibrio cholerae*, *Salmonella* spp. (*Salmonella* Typhi) and *Shigella* spp. (*Shigella flexneri*) in food samples in Bangladesh. Shammi [16] reported contamination of *Vibrio* spp., *Salmonella* spp. and *Shigella* spp. in chicken, beef, fish and shrimp samples collected from local markets of Dhaka, Bangladesh. Mrityunjoy et al. [17] also reported prevalence of *Vibrio cholerae* in different food samples such as chicken, fish, beef and shrimp. Noor et al. [18] reported *Vibrio* spp. and *Shigella* spp. in export oriented shrimp samples of Bangladesh. Fatema et al. [19] reported prevalence of *Salmonella* spp. in poultry meat collected from local markets of Bangladesh. Aktar et al. [20] reported prevalence of *Salmonella* spp. in meat and dry fish samples in Bangladesh. Prevalence of *Vibrio cholerae*, *Salmonella* spp. and *Shigella* spp. in different types of food samples of Bangladesh were reported also by many other researchers [21–24].

This study evaluates microplate IC (wells of 96 well polystyrene plate coated with antibody) as a better alternative to conventional methods for detection of foodborne pathogens.

## Results

**Fig. 1:**
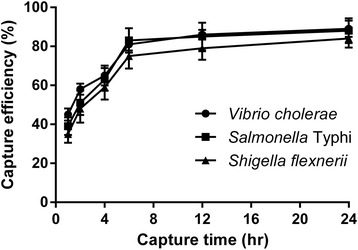
Effect of incubation time on capture efficiency of microplate immunocapture

**Fig. 2:**
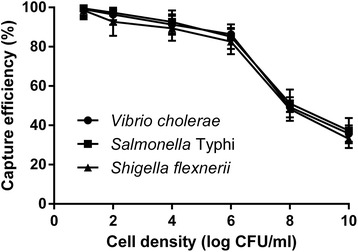
Effect of cell density on capture efficiency of microplate immunocapture

To analyze the capture efficiency of the microplate IC method, bacterial cell was added to the wells and incubated for varying periods (1-24 h). Capture efficiency was increased from 1 h (about 40%) to 6 h (about 80%) gradually, after that it was increased slowly till 24 h (Fig 1). This trend was similar for all three pathogens. Results indicate that 6 h incubation for IC could be considered sufficient to detect the pathogens. To determine optimum inoculum cell density, different concentration of cells was added to IC wells for 6 h and capture efficiency (CE) was determined. Results showed that CE is satisfactory (~80%) up to log6 cfu/ml cell concentration, but at higher cell concentration CE declines rapidly (Fig 2). This may be due to limited space and bound antibody in the wells of 96 well plate. On the basis of the results of Figs. 1 and 2, the optimized cell density and reaction time was selected to be log6 CFU/ml and 6 h respectively.

**Fig. 3:**
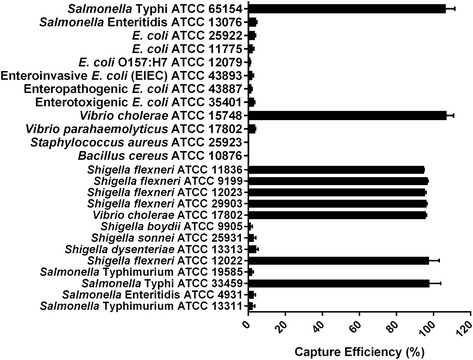
Specificity of microplate immunocapture

To determine the specificity of microplate IC, detection of other related pathogens has been tested. The method showed considerable specificity to detect *Vibrio cholerae*, *Shigella flexneri* and *Salmonella* Typhi (CE was around 100%). CE was zero or very low in case of other bacteria tested (Fig. 3). One of the potential disadvantages of this method is the chance of cross reaction with closely related species, as it occurs in this experiment. Experiments with *Salmonella* Enteritidis, *Salmonella* Typhimurium and *Vibrio parahaemolyticus* showed some CE may be due to cross reaction, though the CE is not significant (*P* < 0.05).

For the detection of the target pathogens in artificially contaminated samples, both IC-culture and IC-PCR showed better sensitivity than the traditional culture method and direct PCR (Table 1). For example, when food samples were inoculated with 10^1^ CFU/25 g bacteria, the IC-culture method can detect *S.* Typhi from 13.3% minced beef samples, *S. flexneri* from 16.7% minced chicken samples, *V. cholerae* from 20% minced fish samples and all the three pathogens simultaneously from 26.7% minced shrimp samples. The IC-PCR method can detect *S.* Typhi from 20% minced beef samples, *S. flexneri* from 16.7% minced chicken samples, *V. cholerae* from 20% minced fish samples and all the three pathogens simultaneously from 33.3% minced shrimp samples. In contrast, the traditional culture method was unable to detect any pathogen in these samples and direct PCR can detect *S.* Typhi from 20% minced beef samples, *S. flexneri* from 11.1% minced chicken samples, *V. cholerae* from 20% minced fish samples and all the three pathogens simultaneously from 20% minced shrimp samples. When food samples were spiked with 10^2^ CFU/25 g bacteria, IC-culture and IC-PCR method can detect the pathogens from more than 30% samples, whereas culture method’s detection range was very low (6.7-13.3%) (Table 1). At higher concentration of bacteria in food (~10^5^ CFU/25 g), the detection ability of the IC and culture method were close (100% in the case of IC-culture and IC-PCR and around 90% in the case of culture method) (Table 1). At all bacteria concentration, IC-culture and IC-PCR method showed better detection performance than direct PCR. The detection ability of the IC (both IC-culture and IC-PCR) method was found to be independent of the origin of the samples. Comparing all the food samples in total, both IC-culture and IC-PCR (can detect pathogens in around 60% of the samples) showed a better sensitivity than the culture method (detect pathogens in around 40% of the samples) and direct PCR (detect pathogens in around 55% of the samples) (Table 1).

**Table 1:** Detection of *Vibrio cholerae*, *Salmonella typhi* and *Shigella flexneri* in artificially contaminated samples

Samples	Pathogen	Method	No of positives/ total no of replicates (%)					Total
			10^1^ CFU/25 g	10^2^ CFU/25 g	10^3^ CFU/25 g	10^4^ CFU/25 g	10^5^ CFU/25 g	
Minced beef (*n* = 5)	*S.* Typhi	IC-culture	2/15 (13.3)	5/15 (33.3)	9/15 (60)	11/15 (73.3)	15/15 (100)	42/75 (56) ^a^
		Culture	0/15 (0)	1/15 (6.7)	5/15 (33.3)	10/15 (66.7)	14/15 (93.3)	30/75 (40)
		IC-PCR	3/15 (20)	6/15 (40)	10/15 (66.7)	13/15 (86.7)	15/15 (100)	47/75 (62.7)
		PCR	3/15 (20)	5/15 (33.3)	9/15 (60)	11/15 (73.3)	14/15 (93.3)	42/75 (56)
Minced chicken (*n* = 6)	*S. flexneri*	IC-culture	3/18 (16.7)	7/18 (38.9)	11/18 (61.1)	15/18 (83.3)	18/18 (100)	54/90 (60) ^a^
		Culture	0/18 (0)	2/18 (11.1)	6/18 (33.3)	11/18 (61.1)	16/18 (88.8)	35/90 (38.8)
		IC-PCR	3/18 (16.7)	9/18 (50)	14/18 (77.8)	17/18 (94.4)	18/18 (100)	61/90 (67.8)
		PCR	2/18 (11.1)	6/18 (33.3)	12/18 (66.7)	15/18 (83.3)	18/18 (100)	53/90 (58.8)
Minced fish (*n* = 5)	*V. cholerae*	IC-culture	3/15 (20)	6/15 (40)	9/15 (60)	12/15 (80)	15/15 (100)	45/75 (60) ^a^
		Culture	0/15 (0)	2/15 (13.3)	5/15 (33.3)	11/15 (73.3)	14/15 (93.3)	32/75 (42.7)
		IC-PCR	3/15 (20)	7/15 (46.7)	11/15 (73.3)	13/15 (86.7)	15/15 (100)	49/75 (65.3)
		PCR	3/15 (20)	7/15 (46.7)	10/15 (66.7)	11/15 (73.3)	13/15 (86.7)	44/75 (58.7)
Minced shrimp^b^ (*n* = 5)	*S.* Typhi *S. flexneri* *V. cholerae*	IC-culture	4/15 (26.7)	7/15 (46.7)	10/15 (66.7)	13/15 (86.7)	15/15 (100)	49/75 (65.3) ^a^
		Culture	0/15 (0)	2/15 (13.3)	6/15 (40)	9/15 (60)	13/15 (86.7)	30/75 (40)
		IC-PCR	5/15 (33.3)	9/15 (60)	11/15 (73.3)	12/15 (80)	15/15 (100)	52/75 (69.3)
		PCR	3/15 (20)	6/15 (40)	9/15 (60)	10/15 (66.7)	11/15 (73.3)	39/75 (52)
MIC = Microplate immunocapture method ^a^Statistically significantly different (*p* = 0.024) according to ANOVA ^b^Only the samples in which all three pathogen detected are included								

Performance evaluation of the microplate IC method with the conventional culture based method showed comparable performance of the method for detection of the pathogens (Table 2). Detection limit of the IC-PCR method (25 CFU/25 g) and IC-culture (50 CFU/25 g) was lower than the culture method (125 CFU/25 g). Detection limit of IC-culture was equal to direct PCR (50 CFU/25 g), but the detection limit of IC-PCR was lower than that of direct PCR. Sensitivity of IC-PCR (100%) and IC-culture (97.3%) was slightly higher than culture method (95.9%), and specificity and accuracy of IC-PCR (97% & 96.2%) and IC-culture (96.1% & 95.3%) was slightly lower than culture method (96.8% & 95.3%) (Table 2). Direct PCR (detection limit- 50 CFU/25 g; sensitivity 98.2%; specificity 95.4%; accuracy 96.1%) has comparable performance to IC-culture (detection limit- 50 CFU/25 g; sensitivity 97.3%; specificity 96.1%; accuracy 95.3%) but the IC-PCR showed better performance (detection limit- 25 CFU/25 g; sensitivity 100%; specificity 96.8%; accuracy 96.7%) than direct PCR.

**Table 2:** Performance evaluation of the microplate immunocapture method as compared to the culture enrichment method

Parameters	Culture	IC-culture	IC-PCR	PCR
Detection limit (CFU/25 g)	125	50	25	50
Sensitivity (CI, 95%)	95.9	97.3	100	98.2
Specificity (CI, 95%)	97.0	96.1	96.8	95.4
Accuracy (CI, 95%)	96.2	95.3	96.7	96.1

## Discussion

Improvements in the microbiological safety of foods have been largely driven by public demand in response to disease outbreaks [25]. The ability to analyze food products for the presence of pathogenic bacteria is essential for verifying the safety of foods, identifying agents of foodborne illness and determining sources of foodborne outbreaks. Conventionally, the microbiological analysis of food involves culture enrichment followed by isolation on selective media [14]. Confirmation of presumptive isolates is generally through biochemical characterization and/or serology. Such methods suffer from a number of drawbacks. In this study, we assessed the suitability of antibody coated 96 well microplates for the detection of three foodborne pathogen, namely *Vibrio cholerae*, *Salmonella* Typhi, and *Shigella flexneri*.

To optimize the microplate IC method for detection of the pathogens, the effect of incubation time on the capture efficiency of the method was determined and results showed that 6 h incubation is sufficient for optimum capture of pathogens from food samples (Fig. 1). The effect of bacterial density on the IC method was also determined and result showed that CE is satisfactory up to log 6 CFU/ml of the target pathogens (Fig. 2). Specificity of the IC method was evaluated and the method showed significant specificity for detection of the target pathogens, *Salmonella* Typhi, *Shigella flexneri,* and *Vibrio cholerae*. The method can discriminate the pathogens from closely related species, too (Fig. 3). Suitability of the IC method for detection of pathogens in food samples was tested with artificially contaminated food samples. Food samples were spiked with different concentration of pathogens, and the IC method and culture method were employed to detect the pathogens. Results of the comparison of IC and culture method have been summarized in Table 1. In the case of minced beef, *S.* Typhi was detected in 40% of samples by conventional culture based method, whereas the pathogen was detected in 56% sample by direct PCR and IC-Culture and IC-PCR method detected *S.* Typhi in 62.7% of the samples. In minced chicken, *S. flexneri* was detected in 38.8% of samples by culture method, in 58.8% of samples by direct PCR, in 60% of samples by IC-Culture, and in 67.8% of samples by IC-PCR. In minced fish samples, *V. cholerae* was detected in 42.7% of samples by culture method, in 58.7% of samples by direct PCR, in 60% of samples by IC-Culture, and in 65.3% of samples by IC-PCR. In the case of minced shrimp, three pathogens (*S.* Typhi, *S. flexneri*, *V. cholerae*) were detected simultaneously and results showed that the pathogens were detected in 40% of samples by conventional culture based method and in 52% sample by direct PCR and in 65.3% of samples by IC-Culture, whereas IC-PCR method detected the pathogens in 69.3% of the samples. Finally, comparison of performance parameters of IC and culture method showed that the IC method offers better performance than the culture method for detection of pathogens from food in terms of the detection limit, specificity, sensitivity, and accuracy (Table 2). The detection limit of IC-PCR (25 CFU/25 g) was shown to be lower than that of the culture method (125 CFU/25 g). Other performance parameters of IC-PCR (sensitivity- 100%, specificity- 96.8%, accuracy- 96.7%) was also improved while compared with culture method (sensitivity- 95.9%, specificity- 97%, accuracy- 96.2%) (Table 2).

Different variations of IC have been developed and reported earlier, most of which are based on immune-magnetic separation (IMS). Xiong et al. [26] reported an IMS method that can capture *E. coli* O157:H7 from food samples with high capture efficiency (>98%) and specificity. Yang [27] reported a di-electrophoresis assisted immune-capture for detection of foodborne pathogens with moderate capture efficiency (~60%). Wang et al. [6] reported an IMS-PCR method for detection of *Alicyclobacillus acidoterrestris* in apple juice has better sensitivity, specificity, and accuracy (90.9%, 97.0% and 96.2%, respectively). Conceicao et al. [28] reported an IMS-Plating method with antibody coated polystyrene microspheres for detection of *Salmonella* sp. in chicken cut with high sensitivity (100%) and specificity (94%).

The microplate immunocapture (MIC) method developed in this study offers a better alternative to the previously reported, IMS methods in terms of ease of operation as it obviates the use of magnetic beads and separation systems. The MIC method also showed competitive performance (sensitivity, specificity and accuracy) with the above mentioned IMS methods. Future research is needed to resolve some shortcomings of this method, such as the effect of high microbial background in food samples. More advanced methods such as whole genome sequencing may provide a more accurate and precise identification scheme for detection of bacteria from food, but in resource-limited laboratories, simpler PCR based method is still the preferable method.

## Conclusion

This study showed that antibody coated microplate can be used for detection of foodborne pathogens from a consortium of non-target organisms with high specificity and sensitivity. This method provides an alternative to the conventional culture based microbiological detection method. The microplate IC method is simple, expensive equipment is not needed and can be modified to detect multiple pathogens simultaneously. From preliminary screening, the method showed promise, but further detailed studies are needed to validate the method for use as a routine method for food laboratories.

## Methods

### Chemicals and reagents

All the media used were purchased from HiMedia (India). Antibodies used were as follows- *V. cholerae* polyvalent antiserum (serovar Inaba & Ogawa); *Salmonella* polyvalent O antiserum; *Shigella flexneri* polyvalent antibody (Denka Seiken, Tokyo, Japan).

### Bacterial strains

*Vibrio cholerae* ATCC-17802, *Salmonella* Typhi ATCC-65154, and *Shigella flexneri* ATCC-12022 were taken from culture collection pool of industrial microbiology laboratory, IFST, BCSIR, Dhaka. The list of other strains used in this study is provided in Table 3.

**Table 3:** List of strains used in this study

List of strains	
For Broth and as reference strain	
	*Vibrio cholerae* ATCC 17802
	*Salmonella* Typhi ATCC 65154
	*Shigella flexneri* ATCC 12022
For specificity test	
	*Shigella boydii* ATCC 9905
	*Shigella sonnei* ATCC 25931
	*Shigella dysenteriae* ATCC 13313
	*Shigella flexneri* ATCC 12022 (serotype-2b)
	*Shigella flexneri* ATCC 29903 (serotype-2a)
	*Shigella flexneri* ATCC 12023 (serotype-4a)
	*Shigella flexneri* ATCC 9199 (serotype-1a)
	*Shigella flexneri* ATCC 11836 (serotype-3)
	*Salmonella enterica* serovar Typhimurium ATCC 19585
	*Salmonella enterica* serovar Typhi ATCC 33459
	*Salmonella enterica* serovar Enteritidis ATCC 4931
	*Salmonella enterica* serovar Typhimurium ATCC 13311
	*Salmonella enterica* serovar Typhi ATCC 65154
	*Salmonella enterica* serovar Enteritidis ATCC 13076
	*E. coli* ATCC 25922
	*E. coli* ATCC 11775
	*E. coli* O157:H7 ATCC 12079
	Enteroinvasive *E. coli* (EIEC) ATCC 43893
	Enteropathogenic *E. coli* ATCC 43887
	Enterotoxigenic *E. coli* ATCC 35401
	*Vibrio cholerae* ATCC 15748
	*Vibrio cholerae* ATCC 17802
	*Vibrio parahaemolyticus* ATCC 17802
	*Staphylococcus aureus* ATCC 25923
	*Bacillus cereus* ATCC 10876

### Preparation of antibody coated microplate

Commercial antibody was diluted 1 to 100 in sterile PBS (10 μl antibody mixed with 990 μl of PBS; working concentration- 10 μg/ml) and 200 μl was added to each well of a polystyrene microplate (Nunc™ Microwell™ 96 μ-well plate, Thermo Scientific, USA) and incubated at 4^0^ C overnight covered with aluminum foil. On the next day, antibody solution was discarded and the microplate was washed 3 times with sterile PBS. For blocking, 200 μl of blocking reagent (1% BSA dissolved in 1X PBS) was added to each well and the plate was incubated for 2 h at 37 °C. After blocking, reagent discarded the wells were washed 3 times with PBS (+10% Tween) solution.

### Capture efficiency (CE)

Capture efficiency was defined as the percentage fraction of the total bacteria captured by the antibodies in the well and was calculated using a method based on the cells unbound in the well or left in the supernatant. Following equation was used for CE calculation-.

CE (%) = (1-B/A)×100%.

Where A is the total number of cells present in the sample (CFU/ml) and B is the number of cells unbound in the well (CFU/ml, in the supernatant and washed solution). Number of unbound cells in the well were counted based on optical density.

For selection of optimum cell density for inoculation and incubation time, capture efficiency was determined with different inoculum cell density (1, 2, 4, 6, 8, and 10 Log CFU/ml) and with capture times ranging from 1 to 24 h (cell density of log 6 CFU/ml).

### Specificity test

To test the specificity as well as the validity of the IC method, and to ensure that the method does not amplify closely related pathogens, a number of closely related and non-target organisms were included in the study (Table 3). The bacteria were cultured in LB broth at 37^0^ C for 12 h, and serially diluted to approximately log6 CFU/ml in PBS containing 1% BSA. Immunocapture was performed and capture efficiency was determined as described above.

### Immunocapture of bacteria

Reference cultures of bacteria were grown in LB broth overnight (condition: temperature- 37^0^ C, shaking- 120 rpm) and enumerated (by drop plate method using nutrient agar media) and cell suspension of different concentrations was prepared. Two hundred microliters of cell suspension was added to respective wells and the plate was incubated at 37^0^ C under shaking condition (100 rpm) for 6 h. A negative control well was used to check cross-contamination. After incubation, the medium was discarded and the wells were washed with sterile PBS and fresh LB broth was added. Bacteria from the well were scraped with sterile loop and streaked onto selective agar plate for respective bacteria (TCBS for *Vibrio cholerae*; SS agar for *Salmonella* and *Shigella*) and the plates were incubated at 37^0^ C overnight. Colonies developed on the agar were sub-cultured onto LB agar and identification was done either by culture method or PCR.

### Isolation of bacteria from food samples

**Fig. 4:**
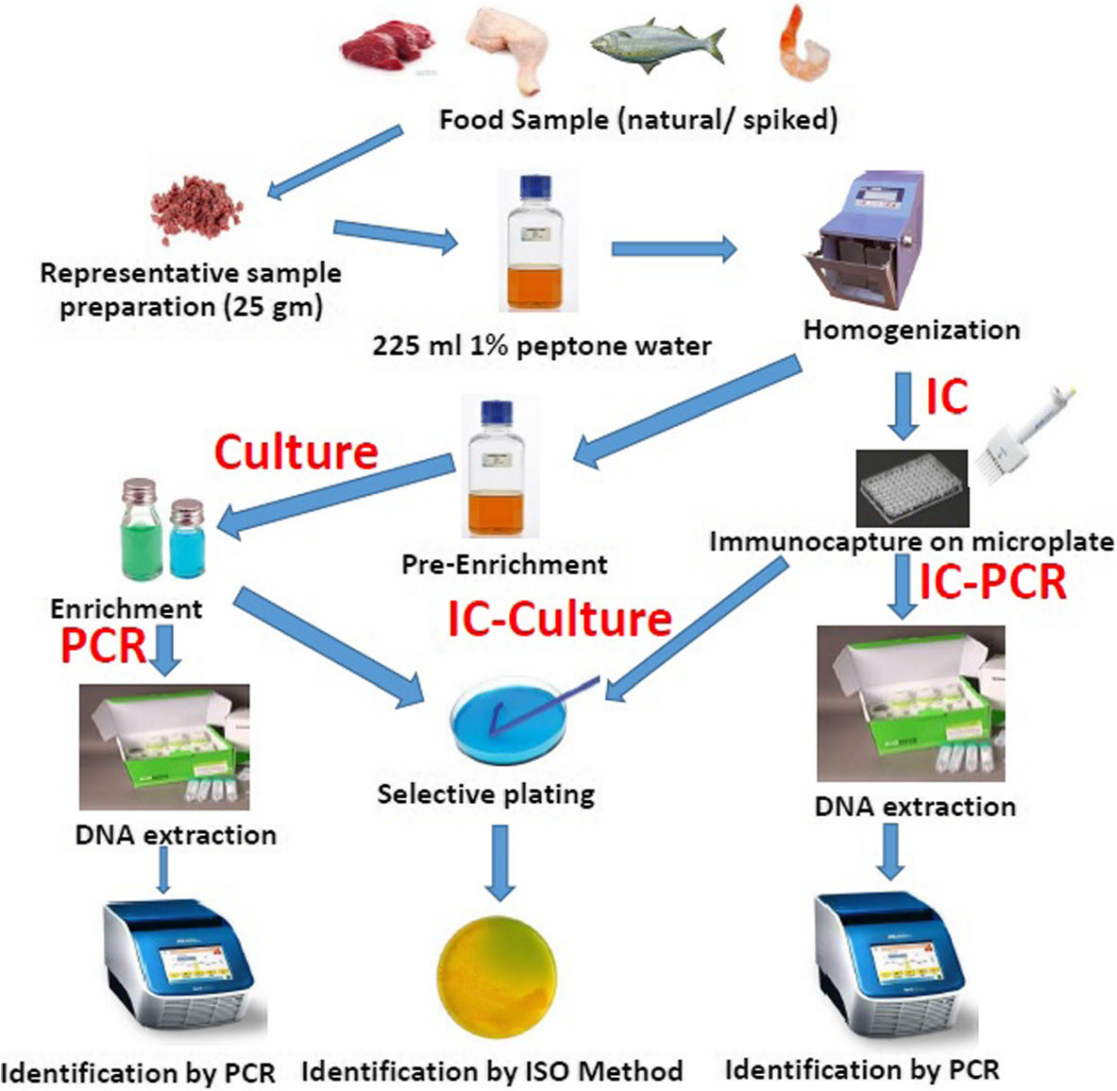
Schematic flow diagram of the experimental protocol used

For spiking of food samples, 250 g of coarsely ground portion of each food type (beef/chicken/fish/shrimp) was used. Spiking was done to obtain different concentration of target bacteria (*S.* Typhi in beef, *S. flexneri* in chicken, *V. cholerae* in fish and *S.* Typhi, *S. flexneri*, *V. cholerae* in shrimp). The Pathogen was grown in LB broth overnight and enumerated and diluted in 10 ml sterile PBS to different concentrations (10^2^ CFU, 10^3^ CFU, 10^4^ CFU, 10^5^ CFU, 10^6^ CFU). Ten 10 ml bacterial suspension was mixed well with 250 g food portion under aseptic condition. Twenty-five grams of representative sample was homogenized in 225 ml of 1% peptone water by stomaching (25 stroke in a sterile bag) (BagMixer®400 W, InterScience, USA) and a part (1 ml) of the stomached sample was used for conventional isolation method with pre-enrichment and enrichment steps. From the enriched samples, 1 ml was used for DNA extraction for detection of pathogen by PCR (hereby referred as direct PCR) and 1 ml was spreaded onto selective media for cultural detection of pathogens. Another portion (1 ml) was used for isolation by IC (both IC-culture & IC-PCR) method (Fig. 4). After IC (as described above), DNA was extracted from microplate well for IC-PCR. For IC-culture, the well was washed with the original media vigorously and the media were inoculated onto selective media by spread plating method. Four types of food samples were used- minced beef (*n* = 5), minced chicken (*n* = 6), minced fish (*n* = 5), minced shrimp (*n* = 5). In case of spiked shrimp sample, all three antibodies were tested together. In other samples, a single antibody was used.

### Identification of bacteria

Identification was done based on ISO methods (ISO 6579-1:2002 [29] for *Salmonella* spp.; ISO/TS 21872-1:2007 [30] for *Vibrio cholerae*; and ISO 21567:2004 [31] for *Shigella* spp.).

For detection of *Salmonella* spp., food sample homogenate in buffered peptone water was pre-enriched at 35^0^ C for 24 h and 1 ml of that was transferred to 10 mls tetrathionate broth and incubated for 24 h at 35^0^ C. The enrichment broth was then plated onto selective media (SS agar and XLD agar) by streaking and the plates were incubated at 35^0^ C for 24 h. In the case of IC-culture, media from each well was spread onto selective media (SS agar and XLD agar) and incubated at 35^0^ C for 24 h. After 24 h, typical *Salmonella* colonies (Black centered colony on SS agar and pink colonies on XLD agar) were selected and identified by biochemical test as described in ISO 6579-1:2002 [29].

For detection of *Vibrio cholerae*, food sample homogenate in buffered peptone water was enriched at 35^0^ C for 24 h. The enrichment broth was then plated onto selective media (TCBS agar) by streaking, and the plates were incubated at 35^0^ C for 24 h. In the case of IC-culture, media from each well was spread onto selective media (TCBS agar) and incubated at 35^0^ C for 24 h. After 24 h, typical *Vibrio cholerae* colonies (large, smooth, yellow and slightly flattened with opaque centers and translucent peripheries) were selected and identified by biochemical test as described in ISO/TS 21872-1:2007 [30].

For detection of *Shigella* spp., food sample homogenate in buffered peptone water was pre-enriched at 35^0^ C for 24 h and 1 ml of that was transferred to 10 mls *Shigella*- broth and incubated for 24 h at 35^0^ C. The enrichment broth was then plated onto selective media (SS agar and XLD agar) by streaking, and the plates were incubated at 35^0^ C for 24 h. In the case of IC-culture, media from each well was spread onto selective media (SS agar and XLD agar) and incubated at 35^0^ C for 24 h. After 24 h, typical *Shigella* spp. colonies (olorless colony on SS agar and red colonies on XLD agar) were selected and identified by biochemical test as described in ISO 21567:2004 [31].

### DNA extraction

Genomic DNA from pure culture of bacteria and from enriched samples was extracted by Accuprep® genomic DNA extraction kit (Cat. No.: K-3032) by the supplied procedure. For extraction of DNA from microplate wells, 200 μl of lysis buffer containing proteinase K was added to each well and the plate was incubated at 70^0^ C for 30 min for lysis. The lysate mixture was transferred to an Eppendorf tube, and 200 μl 100% ethanol was added and mixed prior to centrifugation. After centrifugation, the pellet was washed with 70% ethanol, dried for 15 mins, and resuspended in miliQ water. DNA concentration was measured by Nanodrop spectrophotometer.

### Detection of pathogen by PCR

Primers used for PCR are listed in Table 4. PCR reaction was performed according to the references provided [21–23]. PCR master mix (30 μl) was prepared as follows: sterile de-ionized water-23.2 μl, 10X PCR buffer with Mg-3 μl, 10 mM dNTP mixture- 0.5 μl, forward primer (10 mM)- 1 μl, reverse primer (10 mM)- 1 μl, Taq DNA polymerase- 0.3 μl and template DNA- 1 μl. Each PCR tube containing the appropriate mixtures was heated at 95 °C for 3 min in the thermal cycler (BioRad, USA) to ensure the complete denaturation of DNA templates. The PCR was then continued with the following programs: denaturation for 1 min at 94 °C, annealing for 1 min at 55 °C, and extension at 72 °C, for 1 min. Thirty five (35) cycles of these segments were repeated with a final extension of 10 min at 72 °C. PCR tubes were then stored at −20 °C until further analysis. PCR products were gel electrophoresed in 1% agarose gel for visualization of target band, 470 bp for *sefA* [32], 588 bp for *ompW* [33] and 423 bp for *ipaH* [34].

**Table 4:** Primer sequences and product size of the genes targeted

Gene	Primers	Sequences	Target organism	Product Size (bp)	Reference
*sefA*	A058	5′-GAT ACT GCT GAA CGTAGAAGG-3’	*Salmonella* Typhi	470	[21]
	A01	5′-GCG TAA ATC AGC ATC TGC AGT AGC-3’			
*ompW*	ompWF	5′-·CAC CAA GAA GGT GAC TTT ATT GTG-3’	*Vibrio cholerae*	588	[22]
	OmpWR	5′-GAA CTT ATA ACC ACC CGC G-3’			
*IpaH*	Ipa F	5′- GCTGGAAAAACTCAGTGCCT-3’	*Shigella flexneri*	423	[23]
	Ipa R	5′- CCAGTCCGTAAATTCATTCT-3’			

### Performance evaluation

Sensitivity was determined as TP/(TP + FN) × 100, where TP stands by the number of true positive results and FN is the number of false negative results. Specificity was determined as TN/(TN + FP) × 100, where TN stands for the number of true negative results and FP is the number of false positive results. Accuracy was determined as the number of correct results divided by the number of all returned results.

### Statistical analysis

The study was replicated three times. All the results are expressed as mean ± standard error (SE). The values were compared using the analysis of variance followed by post-hoc multiple comparisons using the Least Significant Difference (LSD) test (v9.1; SAS for Windows, Cary, NC) and differences were considered significant when *P* < 0.05.

## Abbreviation

ATCC: American Type Culture Collection
BSA: Bovine serum albumin
CE: Capture efficiency
CFU: Colony forming unit
IC: Immunocapture
IC-Culture: Immunocapture-culture
IC-PCR: Immunocapture-Polymerase Chain Reaction
IMS: Immune-magnetic separation
ISO: International Organization for Standardization
LB: Luria Bertani
MIC: Microplate Immunocapture
PBS: Phosphate buffered saline
SS: Salmonella-Shigella
TCBS: Thiosulfate citrate bile salt sucrose
XLD: Xylose Lysine Deoxycholate; Min- Minute
